# Perceptions and experiences related to health and health inequality among rural communities in Jimma Zone, Ethiopia: a rapid qualitative assessment

**DOI:** 10.1186/s12939-018-0798-9

**Published:** 2018-06-18

**Authors:** Nicole Bergen, Abebe Mamo, Shifera Asfaw, Lakew Abebe, Jaameeta Kurji, Getachew Kiros, Muluemebet Abera, Gebeyehu Bulcha Duguma, Kunuz Haji Bedru, Manisha A. Kulkarni, Ronald Labonté, Sudhakar Morankar

**Affiliations:** 1 University of Ottawa, 600 Peter Morand Crescent, Ottawa, ON K1G 5Z3 Canada; 2 Jimma University, PO Box 378, Jimma, Ethiopia; 3 Jimma Zonal Health Department, Jimma, Ethiopia

**Keywords:** Social determinants of health, Health equity, Meaning of health, Maternal and child health, Qualitative methods

## Abstract

**Background:**

The Safe Motherhood Research Project studies the implementation and scale-up of maternal, newborn and child health (MNCH) initiatives in Jimma Zone, Ethiopia. This qualitative rapid assessment study was undertaken to explore community perceptions and experiences related to health, health inequality and other MNCH themes.

**Methods:**

We conducted 12 focus group discussions and 24 in-depth interviews with community stakeholder groups (female and male community members, Health Extension Workers, members of the Women Development Army and Male Development Army, and religious leaders) across six rural sites in Jimma Zone. Data were analyzed through thematic coding and the preparation of content summaries by theme.

**Results:**

Participants described being healthy as being disease free, being able to perform daily activities and being able to pursue broad aspirations. Health inequalities were viewed as community issues, primarily emanating from a lack of knowledge or social exclusion. Poverty was raised as a possible contributor to poor health, however, participants felt this could be overcome through community-level responses. Participants described formal and informal mechanisms for supporting the disadvantaged, which served as a type of safety net, providing information as well as emotional, financial and social support.

**Conclusions:**

Understanding community perceptions of health and health inequality can serve as an evidence base for community-level initiatives, including MNCH promotion. The findings of this study enable the development of audience-centered MNCH promotion activities that closely align with community priorities and experiences. This research demonstrates the application of rapid qualitative assessment methods to explore the context for MNCH promotion activities.

## Background

Since the mid-1990s, the health sector in Ethiopia has undergone major transformations, changing the landscape of how health services are delivered by prioritizing community-based interventions that focus on maternal, newborn and child health (MNCH) and disease prevention in underserved populations [1–3]. Notably, through the training and deployment of paid community health workers (Health Extension Workers (HEWs)), the country’s Health Extension Program has been successful in reaching rural communities and improving several health indicators [4–6]. For instance, the percentage of women who received any antenatal care from a skilled provider increased from 27% in 2000 to 62% in 2016; over the same period, the percentage of live births delivered in a health facility has increased from 5 to 26% [7]. Despite remarkable improvements to overall health in Ethiopia [8], the country continues to face large health inequalities [9] and halted progress in certain development markers [10]. For instance, the underutilization of MNCH services constitutes a major challenge, with barriers stemming from delays in the decision to seek health care, inability to access a health facility, and failure to receive timely and appropriate medical treatment [11–13].

The Ethiopian Ministry of Health has recognized the need for a revitalized approach to health promotion that: develops context-specific health program communication strategies, builds upon detailed situational analyses, and accounts for community-level diversity [14]. In low-resource settings such as rural Ethiopia, however, community-level evidence is often limited in scope, dated or lacking altogether.

This study was undertaken during the initial phase of a five-year (2015–2020) research project, The Safe Motherhood Project,^1^ to inform the design and delivery of its two MNCH promotion interventions: information, education and communication activities in rural communities, and improved infrastructure and functionality of maternal waiting areas at rural health facilities. This study aims to deploy a rapid qualitative assessment approach to better understand community stakeholders’ perceptions and experiences with regards to health, health inequalities and MNCH. As applied in other studies, *perception* refers to how people understand a phenomenon and its causes and effects, which in turn, influences how they experience the phenomenon [15]. In our study, we explore stakeholder perceptions of health and health inequality generally, and also drawing from their experiences (personal encounters) related to MNCH. In this article, we report on findings from community members and groups across three districts of Jimma Zone. While this exploration has implications for health promotion activities across various health topics, the findings of the research are primarily considered in their application to MNCH promotion.

## Methods

### Sampling and participant recruitment

The study was conducted at six rural health posts, located in the three districts of Jimma Zone. A convenience sample of two kebeles (communities of about 5000 people served by one health post) per district participated; the criteria for kebele selection included location (proximity to Jimma and/or passable roadways), previous contact with personnel at the health post, and willingness to participate in the study. We conducted 12 focus group discussions (FGDs) with 6–12 participants, and 24 semi-structured, in-depth interviews (IDIs). FGD participants included female and male community members. HEWs, members of the Women Development Army (WDA),^2^ members of the Male Development Army (MDA),^3^ and religious leaders all participated in IDIs. Participant recruitment for the FGDs and IDIs was done through the HEWs at each kebele health post.

### Data collection

Data were collected in the local language (Afan Oromo) using semi-structured FGD and IDI guides. The guides were prepared in English by the research team, translated into Afan Oromo, and pilot-tested in an area that was not included in the study. The FGDs and IDIs included questions about demographics, what it means to be healthy, factors that explain why some are healthier than others, and feelings/responses to inequalities in health. The IDIs with community stakeholders additionally asked participants about their role in promoting health, and related barriers and enablers. Nine bilingual individuals (seven males and two females) from Jimma University were employed to assist with data collection, and participated in a two-day induction workshop.

Data collection took place over six weeks in May–June 2016. FGDs were facilitated by teams of two data collectors, and there was one data collector per IDI. A member of the research team obtained written or oral informed consent from all participants. Data collectors worked independently, and at least two researchers with the Safe Motherhood Project were present in a supervisory capacity. Daily debriefing sessions with the researchers and data collectors were held to discuss key findings, refine the FGD and IDI guides, and identify strategies to enhance data collection. To document their experiences and impressions, data collectors and researchers kept field notes, and researchers conducted an exit interview with each data collector to further probe the content of the field notes and debriefing sessions. All FGDs and IDIs were recorded and transcribed into English by the assigned data collector. A random sample of transcripts was checked against the recordings by a bilingual researcher.

### Data analysis

An initial code guide was developed by two of the researchers (NB and AM), and expanded to incorporate emergent ideas from an initial selection of transcripts. Following multiple readings of all transcripts, a final version of the code guide was developed through consensus by NB and AM. The final code guide allowed us to capture all major ideas raised by the participants in each thematic category. Transcript coding was done in Atlas.ti software (version 7.5.12) by three researchers (NB, AM and SA). To enhance inter-coder reliability, the researchers independently applied the code guide to a selected transcript, and then reviewed and resolved any differences. Reports of quotations for selected codes were generated in Atlas.ti software, and the researchers prepared content summaries of findings.

### Ethical clearance

Ethical clearance for this research was obtained from Jimma University College of Health Sciences Institutional Review Board and University of Ottawa Health Sciences and Science Research Ethics Board; the research was conducted in accordance with the prevailing ethical principles.

## Results

### Meaning of health

Across all participant groups, health was frequently referred to as an asset that was a prerequisite to living, thriving and deriving pleasure from life. Overall, participants emphasized the importance of having good relationships within the larger community, supporting each other emotionally and financially, and working with different sectors of the community (e.g. the WDA, MDA, religious leaders, HEWs and other bodies). Belonging to a community enabled individuals to be healthy, as the community collectively encouraged and advocated for one another’s health. One male community member expressed how community relationships were related to his own health condition:

“I am healthy if I live in peace, when my mind and my body parts are free from problems, when my family is in good condition, and when I have good relationships with my community. When I live a favourable life, I can say I am a good and healthy man.”

-Male community member, Gomma district, age 49.

#### Biomedical concepts

Participants expressed that being healthy meant being free from disease and disease symptoms, citing examples such as AIDS, malaria, diarrhea, the common cold and gastritis; additionally, individuals who had mental health issues or physical disabilities were not considered to be healthy. Participants mentioned the use of both preventive and curative measures to avoid or mitigate disease. These included: following the advice of health workers, getting treated for ailments, taking medication as directed, and using available health services and facilities. For instance, a male community member from Kersa district (age 40 years) credited the use of health services and facilities for the better health of his second child.

#### Ability to perform daily activities

Many participants spoke about health as the capacity for physical functioning – the ability to complete daily activities, walk and move freely, and be productive. A female community member explained:

“A healthy person is someone who has functional hands and legs, who has a peaceful mind, who is able to produce and eat, who is able to move to where he wants, and who is able to learn and produce. Unless he is healthy, he can’t accomplish anything or produce for survival.”

-Female community member, Kersa district, age 25 years.

For women, key daily activities included taking care of their children, preparing food and maintaining the household. For men, daily activities were related to working and ensuring the livelihood of the family (e.g. ability to plough the fields). Religious leaders, HEWs and members of the WDA and MDA made references to daily activities related to their respective roles within the community.

#### Opportunities for advancement

Participants across several study sites related health with notions of peace, security, education, wealth, and development. In general, participants perceived that health is a prerequisite for broader, societal aspirations, and a community must achieve a certain threshold of health among its members to live in harmony and advance. For example, the health status of the community was linked to the ability of its members to protect themselves, support one another during happy and sad times, and secure sufficient wealth to provide for their families. The relationship between health and peace was described by one man as:

“Health is peace; if there is no peace, not all people are healthy. In the community, if there is health but no peace, it is difficult to do our daily activities like keeping our personal and environmental hygiene as well as keeping our children clean and healthy. So health and peace have a strong relationship.”

-Male community member, Gomma district, age 40 years.

### Perceptions of health inequality

Participants acknowledged that not everyone is in good health, even within the same community. Participants generally believed they had agency over their health status, though certain conditions were viewed as unavoidable, such as congenital disabilities attributed to the “will of God”. Inequalities in health were largely viewed as community issues: although they sometimes traced back to individual characteristics or behaviours, the community was ultimately responsible for supporting individuals in achieving and/or maintaining health.

#### Root causes of disadvantage

Participants highlighted three main reasons why some people were less healthy than others. First, individuals may lack awareness or knowledge in areas such as how to practice safe sex, how to keep themselves and their homes clean, and how to eat nutritiously. Moreover, participants emphasized the importance of understanding how and when it is appropriate to use health services and/or take medication. One male community member explained:

“People have problems in taking their sick families to health facilities. Some wait longer before they take their family members to the health facilities. This emanates from a lack of awareness. The Health Extension Worker tries what she can, but the people do not have awareness.”

-Male community member, Kersa district, age 35 years.

Second, social exclusion – a state where people or groups are excluded from social systems or relationships [16] – may lead to poor health. Individuals who are outsiders in the community do not have access to the support structures that promote and enable healthy behaviours. Further, those in conflict with others, or who lack love from others, suffer health problems. One community member explained the importance of addressing loneliness, especially among the poor:

“What matters is loneliness. We have to convince everyone to live together because living together is an asset itself. If the poor live alone, they lack social support. We can support them, then in any situation, a person has an asset. Even for the rich, living alone is not good.”

-Female community member, Kersa district, age 25 years.

Financial poverty was cited as a third factor that could result in some individuals having worse health than others. Being poor made it difficult to gain access to resources and services required to lead health lives. A female community member provided an example of how not having money was a barrier to securing better living conditions, which in turn, compromised health:

“There are some diseases like cough. If we relate this with wealth, a poor family that lives in small unventilated house can’t prevent the cough from spreading because of overcrowding. If they had money, they could build a house with ventilated windows.”

-Female community member, Kersa district, age 22 years.

Being poor also made individuals feel worried and stressed, putting them at risk for mental health problems. Several participants, however, explained that the negative effects of poverty could be overcome by being knowledgeable about health and/or having access to social supports; that is, having financial resources did not guarantee better health unless those resources were used in a way that promoted health.

#### Community responses to health inequality

When questioned about what should be done to address health inequalities, participants usually identified responses at the community level. A collectivist approach to addressing problems was evident in a proverb quoted by a participant:

“*Namnni tokko yoo dhukkubsate yoo namatti himate dawaa argata*. This means that a person could get relief for his problem only if he told it to others. Because of this, our community networks get involved to share the problem and help each other to encourage the person to visit a health facility. It is better to help each other to live in this world.”

-Female community member, Seka Chekoresa district, age 28 years.

Participants described formal and informal mechanisms for supporting the disadvantaged, which serve as a type of safety net, providing information, as well as emotional, financial and social support. Formal mechanisms included health insurance programs (implemented at some study sites), a program that identifies model households, and the one-to-five networks. Informal mechanisms varied across study sites. For instance, a female community member from Kersa district identified two groups in her community:

“We have two women organizations: the support group *garee wal-gargaarsaa* and the money saving group *garee qusannoo*. Using these organizations, we can help. We use the money collected if the individual with problems are poor. If the person has no awareness, we have to advise, be with him and encourage him to take all of the prescribed medicine. And we support them emotionally. So, it’s possible to use either organization to help a person in trouble.”

-Female community member, Kersa district, age 26 years.

HEWs, religious leaders and members of the development army identified aspects of their respective roles that served to promote health in their communities, often with intersecting roles that encouraged a community approach. A religious leader, for example, explained how he encouraged his followers to support those in need:

“When someone from the community becomes ill, the followers have a responsibility to help that person by taking him to the health facility as well as by supporting him economically until he improves. So, it is our responsibility and we [religious leaders] teach our followers about their duties.”

-Religious leader, Kersa district, age 39 years.

## Discussion

### Key findings and contributions

The findings of this study add to the current body of literature in two important ways. First, our exploration the general meaning of health and health inequalities provides a scoping overview of the context for the Safe Motherhood Project, extending beyond the specific topic of MNCH. This is a valuable contribution, as no previous studies, to our knowledge, have published this type of community-level, empirical research in rural Jimma Zone. To date, needs assessment activities in Ethiopia have tended to adopt a vertical focus, capturing a narrower range of health topics. Further, previous research has called attention to key differences between areas throughout the country related to how communities experience delays in receiving MNCH care [13].

Second, our application of qualitative methods in a community setting is a novel approach to explore the context for MNCH promotion activities, especially in rural Jimma Zone. Our results complement and extend findings of previous studies, which have tended to rely more heavily on quantitative-based survey methods. For instance, one study, undertaken as baseline research for the Communication for Health Project (covering four regions in Ethiopia, including Oromia region where Jimma Zone is located), reported lower MNCH service use among women with high vulnerability scores, which were constructed based on food, shelter and monetary resources [17]. The qualitative methods employed in our study allowed us to probe deeper about the notion of vulnerability, highlighting the risks associated with lack of awareness and social exclusion. We also gained insight into diverse formal and informal community mechanisms to encourage and enable health service use.

Several other studies have administered structured questionnaires to individuals in Jimma Zone on topics related to reproductive health, with the aim of identifying knowledge gaps, information sources and/or provider preferences [18–20]. Our results illustrate the rich context in which such knowledge, attitudes and practices develop and are reinforced. A more complex appreciation for how and why health is valued, and the benefit that good health brings to a community, can serve as considerations in the planning phases of research and/or program development and implementation. For example, efforts to promote facility births may be strengthened by harnessing the support of the community in referring and/or transporting rural and remote women to facilities; to this end, initiatives such as maternal waiting areas (residences near health facilities that accommodate women in their final stages of pregnancy [21]) may be positioned to achieve greater reach if promoted by the community.

In this study, the importance of community was a predominant theme that shaped participants’ understandings of health and health inequalities. This was unsurprising, as Ethiopia is generally considered a highly collectivist society that emphasizes relationships, group obligation, and interpersonal harmony [22]. Like our study, other research in Ethiopia has reported a diversity of perceptions and experiences on health matters [23]. Our study, however, revealed that responses were more similar by setting than by stakeholder role; that is, community-level interests and experiences were prioritized over individual roles. Even participants that convened regularly at more centralized levels (e.g. HEWs and religious leaders) did not necessarily express shared ideas.

Participants in this study expressed that, as a community, they experienced a strong sense of agency over their health. Community networks that pooled/redistributed money and provided social support were ubiquitous across study sites. Generally, participants felt that most of the resources necessary for good health were already available within the community, and that health could be improved by sharing or mobilizing those resources. For example, HEWs were viewed as a trusted source of information about health, and an access point to obtain basic services or referrals to higher levels of the health system, which has also been reported elsewhere in Ethiopia [24–27]. Others, however, have highlighted the top-down nature of Ethiopia’s Health Extension Program, and questioned the extent to which local health workers are expected to remain subordinate [28]. Indeed, social desirability tendencies and the complexity of situating health interventions within specific local cultural and social forces remain important considerations that further support the need for contemporary, context-specific research.

### Strengths and limitations

This research benefited from the use of semi-structured FGDs and IDIs, which allowed for open-ended questioning and probing, and generated rich descriptions of community perceptions of health and health inequality. The inclusion of multiple and diverse community stakeholders across six study sites enriched the breadth of the data, and allowed for exploration of how themes emerged across and between study sites. Another strength of this study was the collaborative approach to working with data collectors, whose impressions and observations helped the researchers to understand and situate the findings, and enhanced the transparency and trustworthiness of the research.

Possible limitations included the method of participant recruitment (through the HEWs), which may have led to the selection of participants that would speak favourably about the HEWs. This recruitment method was used because HEWs served as the main contact between the research team and the community, and the HEWs were able to access participants through their community networks. Other researchers, however, have acknowledged that political context has important implications on health research methodology [29]. In effort to account for and minimize potential bias, we worked closely with the data collectors before and during data collection to develop strategies to encourage participants to speak freely. These included: providing participants with detailed explanations of measures to ensure confidentiality and anonymity; conducting FGDs and IDIs in private areas; and using question probes and different entry points to elicit responses (e.g. use of shadowed data where participants were asked to refer to other’s experiences in addition to their own [30]). Additionally, field notes and data collector exit interviews were read alongside transcripts during data analysis to add depth and context.

## Conclusions

Our approach to doing a rapid assessment of the meaning of health and health inequalities in rural Jimma Zone upholds principles outlined by the National Health Promotion and Communication Strategy for Ethiopia, and supports the community-level action in the Strategy’s framework [14]. The findings of this research enable the development of audience-centered health promotion initiatives, such as MNCH promotion through the Safe Motherhood Project.

Moving forward, the MNCH promotion activities of the Safe Motherhood Project will foster a sense of community ownership and participation, as key messages will be closely aligned with existing community systems, priorities and experiences. The findings from these data are positioned to serve as an evidence basis from which to monitor and improve the health promotion activities of the Safe Motherhood Project, as well as other initiatives by the Zonal and Regional Health Bureaus.

## Abbreviations

FGD: focus group discussion
HEW: Health Extension Worker
IDI: in-depth interview
IEC: information, education and communication
MDA: Male Development Army
MNCH: maternal, newborn and child health
WDA: Women Development Army

## Footnotes

1 The Safe Motherhood Project is an intervention trial that supports the implementation and scale-up of MNCH initiatives in selected districts of Jimma Zone. For more information about the Safe Motherhood Project, please see: https://www.idrc.ca/en/project/promoting-safe-motherhood-jimma-zone-ethiopia-imcha.

2 The Women Development Army is a voluntary community health workforce that supports the work of Health Extension Workers. One women out of every five households is a Women Development Army member, responsible for promoting health among the five households under the supervision of Health Extension Workers.

3 The Male Development Army has a similar structure to the Women Development Army, with a greater focus on aspects of community development related to physical infrastructure or agriculture.
